# Chronicling menstrual cycle patterns across the reproductive lifespan with real-world data

**DOI:** 10.1038/s41598-024-60373-3

**Published:** 2024-05-03

**Authors:** Adam C. Cunningham, Lubna Pal, Aidan P. Wickham, Carley Prentice, Frederick G. B. Goddard, Anna Klepchukova, Liudmila Zhaunova

**Affiliations:** 1 Flo Health UK Limited, 27 Old Gloucester Street, London, WC1N 3AX UK; 2 Department of Obstetrics, Gynecology and Reproductive Sciences, Yale School of Medicine, New Haven, CT USA

**Keywords:** Digital health, Health literacy, Menstrual cycle, Period tracking app, Women’s health, Reproductive aging, Medical research, Reproductive biology

## Abstract

The intricate hormonal and physiological changes of the menstrual cycle can influence health on a daily basis. Although prior studies have helped improve our understanding of the menstrual cycle, they often lack diversity in the populations included, sample size, and the span of reproductive and life stages. This paper aims to describe the dynamic differences in menstrual cycle characteristics and associated symptoms by age in a large global cohort of period-tracking application users. This work aims to contribute to our knowledge and understanding of female physiology at varying stages of reproductive aging. This cohort study included self-reported menstrual cycle and symptom information in a sample of Flo application users aged 18–55. Cycle and period length and their variability, and frequency of menstrual cycle symptom logs are described by the age of the user. Based on data logged by over 19 million global users of the Flo app, the length of the menstrual cycle and period show clear age-associated patterns. With higher age, cycles tend to get shorter (Cycle length: ${\overline{\text{D}}}$ = 1.85 days, Cohen’s D = 0.59) and more variable (Cycle length SD: ${\overline{\text{D}}}$ = 0.42 days, Cohen’s D = 0.09), until close to the chronological age (40–44) suggesting menopausal transition, when both cycles and periods become longer (Cycle length: ${\overline{\text{D}}}$ = 0.86 days, t = 48.85, Cohen’s D = 0.26; Period length: ${\overline{\text{D}}}$ = 0.08, t = 15.6, Cohen’s D = 0.07) and more variable (Cycle length SD: ${\overline{\text{D}}}$ = 2.80 days, t = 111.43, d = 0.51; Period length SD: ${\overline{\text{D}}}$ = 0.23 days, t = 67.81, Cohen’s D = 0.31). The proportion of individuals with irregular cycles was highest in participants aged 51–55 (44.7%), and lowest in the 36–40 age group (28.3%). The spectrum of common menstrual cycle-related symptoms also varies with age. The frequency of logging of cramps and acne is lower in older participants, while logs of headache, backache, stress, and insomnia are higher in older users. Other symptoms show different patterns, such as breast tenderness and fatigue peaking between the ages of 20–40, or mood swings being most frequently logged in the youngest and oldest users. The menstrual cycle and related symptoms are not static throughout the lifespan. Understanding these age-related differences in cycle characteristics and symptoms is essential in understanding how best to care for and improve the daily experience for menstruators across the reproductive life span.

## Introduction

Menstruation, and menstrual health concerns, are severely underrepresented in the health research landscape^1^. The female reproductive system ages faster than other systems in the body^2^. This has profound implications for the menstrual cycle, fertility, and the functioning of other organ systems and ultimately leads to the female reproductive lifespan being shorter than the average expected lifespan of the individual. The characteristics of the menstrual cycle, like cycle length and regularity, and the profile or severity of menstrual cycle related symptoms such as weight gain, sleep problems, or headaches are highly age-dependent^3^.

Aside from cycle and period length variability due to health conditions or other environmental exposures, it is known that cycle length (the interval in days between the onset of a menstrual bleed and the onset of the subsequent menstrual bleed) and period length (days of bleeding) tend to shorten and become more variable with age until the final menstrual period at menopause, when the menstrual cycle ceases completely^4^. However, this linear overview of menstruation and age may be too simplistic and can disguise more subtle age-related effects, particularly early and late in the reproductive life span, such as an increase in cycle length after age 50, in the years immediately before the menopause^5^. While large-scale studies exist, they have often relied on participants recruited from specific settings, such as healthcare^6^, relatively narrow age ranges^7^, or specific countries or geographies^6–10^. Often, participants older than 40 are excluded from research into characteristics of the menstrual cycle across the spectrum of reproductive aging. Our current understanding of both menstrual cycle patterns, and the related symptomatology experienced by menstruators across the spectrum of reproductive aging (from early adulthood into perimenopause) has been shaped by studies that are limited in sample size and restricted in the scope of population representation. Thus, there is a need for large-scale research investigating the cycle and related symptoms in naturalistic samples across the reproductive life span.

Alongside differences in the length and variability of the menstrual cycle with advancing age, there are also differences in the menstrual cycle-related symptoms experienced by individual menstruators. For example, breast tenderness is most common in the teenage years, but most severe in the late 30s and early 40s^11^. Reports of sleep disturbances tend to increase with advancing age, particularly during the phase of menopausal transition and early menopause^12^. Indeed, the transition to menopause is associated with a range of symptoms including hot flashes, mood changes, impaired cognition, and sleep disturbance among others^13^. Around 60–80% of women transitioning towards and into menopause experience classic menopause symptoms that are severe enough to lead to many seeking medical care^13–15^. Regardless of life stage, the average human female experiences approximately 400 cycles over their lifetime^16^, meaning that any disruptive menstruation-related symptoms can lead to a significant health and quality of life burden.

Despite efforts to improve menstrual health literacy, understanding of what constitutes a “normal” menstrual cycle, both from the perspectives of menstruators and healthcare professionals remains wanting^16^. For example, irregular length cycles may be expected in individuals beyond age 45 and regarded as a symptom of the transition to menopause, but would be a reason for medical investigation in younger individuals. Furthermore, many women lack clarity on when they should seek medical help for menstrual cycle-related symptoms such as dysmenorrhea, which affects around 45–95% of menstruating individuals, depending on the classification criteria used^17^, or for heavy menstrual bleeding, which has a prevalence of around 27–39%^18^. Strikingly, it has been suggested that as few as 1 in 10 women in the UK felt they had received enough information about what to expect during the transition to menopause^19^. Improving our knowledge and understanding of the variability in symptoms experienced across the reproductive lifespan is necessary to help improve menstrual health literacy.

There are now many smartphone applications (mobile health *apps* [mHealth *app]*) available, and many are increasingly being developed and used to track the menstrual cycle in order to gain clarity on menstrual patterns and symptoms. These *apps* allow users to input data well beyond the actual menstrual events, including data on physical and mood symptoms, sexual activity, and physical activity. Tracking the menstrual cycle can help users understand their physiology, and better plan for their biological goals as well as promote healthy habit forming or behavior^20–22^.

Flo (by Flo Health UK Limited) is a health, well-being, and period tracker mHealth app for women and people who menstruate, with over 58 million monthly active users. The Flo *app* (https://flo.health/) offers artificial intelligence (AI) based period prediction, allowing users the ability to track their periods, fertile window, and timing of ovulation, as well as record a wide variety of physical and psychological symptoms. A unique aspect of The Flo *app* is that it allows users access to a large library of evidence-based educational content relating to menstrual and reproductive health, information that has been vetted by medical and scientific experts in the field of women’s health. The real-life data that can be collected through *apps* such as Flo offers the potential to serve as a rich resource for population health studies into the menstrual cycle and its symptoms, reproductive wellness, and disorders using ‘digital epidemiology’ approaches^23–25^.

In this paper, we aim to describe age-related differences in menstrual cycle length, bleeding length, and symptoms logged by a sample of women and people who menstruate aged 18–55 who have tracked their menstrual cycle and symptoms using the Flo *app*.

## Methods

This study included global users of the Flo *app* who recorded bleeding days or symptoms in a 12-month period between the 11th of December 2022 and the 11th of December 2023. Users were between 18 and 55 years of age at the time of data extraction, had not recorded a pregnancy during the 12-month period for which data was extracted, in order to avoid additional noise in the cycle data due to disruption of the cycle during and after pregnancy, and had recorded at least three menstrual cycles. Menstrual cycles that were shorter than 10 days were excluded as cycles with an unrealistic cycle length. To attempt to target users not on hormonal contraception, users who had set contraception reminders were also excluded. Cycle and period lengths were calculated based on self-reported user data. Users report the start and end dates of their period, with the cycle start date defined as the first day of bleeding. The period was defined as the number of days from the first day of bleeding until the last day of bleeding. If a user logs the start of bleeding but does not provide an end date, the bleeding length is automatically set to five days. Participants provided informed electronic consent for aggregated data that could not identify them as individuals to be used for research purposes when agreeing to the usage terms and Privacy Policy of the Flo app. The study protocol was approved by an independent ethics review board (Western Copernicus Group Independent Review Board—WCG IRB, number 20226050).

Symptoms of interest were self-reported and included somatic (Cramps, Tender Breasts, Headache, Fatigue, Backache, Acne), gastrointestinal (Bloating, Constipation, Diarrhea, Nausea), mood (negative [Mood swings, Anxious mood, Depressed mood, Confused mood, Insomnia, Stress, Obsessive Thoughts], positive [Energetic mood, Happy]) and vaginal discharge (Sticky, Egg white, Creamy, Watery). Flo *app u*sers are able to record as many symptoms in each category as desired each day.

In addition to the cycle and symptom data, self-reported information on body mass index (BMI, in kg/m^2^) and current age (in years) at the time of data extraction were also obtained for each user.

### Statistical methods

Individual mean and standard deviations of cycle length and period length were calculated for each user. The standard deviation of cycle length was used to assess cycle variability. No outlier removal other than excluding cycles shorter than 10 days was performed as we expect longer and more irregular cycles in older age groups. These mean and standard deviation values were then aggregated by age group (18–25, 26–30, 31–35, 36–40, 41–45, 46–50, or 51–55), to facilitate comparisons between age groups. A one-way ANOVA was used to compare differences between mean lengths and standard deviations between age bands, while T-tests were used to compare differences between mean lengths and standard deviations between specific age groups. In addition, the mean cycle or period length and mean standard deviation of cycle or period length were plotted by age to visualize age-associated differences in these outcomes.

Flo *app* users were classed as having irregular cycles if the difference between the longest and shortest cycle recorded within the time period of interest was greater than 7 days at least twice in the 12-month study period.

To investigate the frequency of symptom logging by age, the mean number of symptom logs for each symptom type was plotted by age. For the symptom analysis, users who recorded more than five times the standard deviation of the mean of the total logs per user were excluded from the symptom level analysis due to having an extremely high rate of logging.

Due to the very large sample sizes used in these analyses, we do not report p-values. In large samples, p-values can be very small even if the difference between groups are very small, and not clinically useful or significant^26^. Instead, we report mean differences and effect sizes where relevant.

All analysis was performed in R version 4.3.0^27^ and the following R packages: arrow v. 12.0.1^28^, arsenal v. 3.6.3^29^, gtsummary v. 1.7.1^30^ mgcv v. 1.8.42^31^, tidymv v. 3.4.2^32^, pwr v. 1.3.0^33^, patchwork v. 1.1.2^34^, tidyverse v. 2.0.0^35^ and rstatix v. 0.7.2^36^.

### Ethical standards

The authors assert that all procedures contributing to this work comply with the ethical standards of the relevant national and institutional committees on human experimentation and with the Helsinki Declaration of 1975, as revised in 2008. The study protocol was approved by an independent ethics review board (Western Copernicus Group Independent Review Board—WCG IRB, number 20226050).

## Results

This study included 19,266,573 users aged 18–55 who had recorded at least three cycles longer than 10 days, had not used the pregnancy mode in the data extraction period, and had not set reminders for contraception. Most users were in the 18–25 age group (N = 8,757,345), with a lower representation of users for each age group that follows (Table 1). Most users had a BMI of between 18.5 and 24.9.

**Table 1:** Summary statistics for the users included in the study.

	All ages, N = 19,266,573	18–25 years, N = 8,757,345	26–30 years, N = 3,822,121	31–35 years, N = 2,948,002	36–40 years, N = 2,055,795	41–45 years, N = 1,234,400	46–50 years, N = 412,761	51–55 years, N = 36,149
BMI, n (row %)								
< 18.5	1,473,977 (18%)	879,766 (21%)	267,006 (17%)	167,607 (15%)	96,897 (14%)	48,468 (13%)	13,160 (12%)	1073 (11%)
18.5–24.9	4,217,269 (53%)	2,317,573 (56%)	818,571 (51%)	541,616 (49%)	320,514 (47%)	165,288 (45%)	49,612 (43%)	4095 (43%)
25–29.9	1,379,175 (17%)	589,918 (14%)	296,437 (19%)	224,131 (20%)	148,541 (22%)	88,091 (24%)	29,489 (26%)	2568 (27%)
30–34.9	557,407 (6.9%)	210,220 (5.1%)	124,529 (7.8%)	99,458 (9.0%)	67,469 (9.9%)	40,990 (11%)	13,571 (12%)	1170 (12%)
35–39.9	221,927 (2.8%)	78,710 (1.9%)	50,894 (3.2%)	41,950 (3.8%)	28,311 (4.1%)	16,520 (4.4%)	5,105 (4.5%)	437 (4.6%)
≥ 40	171,261 (2.1%)	61,429 (1.5%)	39,641 (2.5%)	32,951 (3.0%)	21,619 (3.2%)	12,016 (3.2%)	3,351 (2.9%)	254 (2.6%)
Unknown	11,245,557	4,619,729	2,225,043	1,840,289	1,372,444	863,027	298,473	26,552
Cycle length in days, mean (SD)								
Mean cycle length	28.54 (3.24)	28.99 (3.31)	28.76 (3.23)	28.28 (3.11)	27.68 (2.96)	27.18 (2.81)	27.15 (2.94)	28.00 (3.70)
Standard deviation of cycle length	4.01 (4.74)	4.14 (4.73)	3.95 (4.81)	3.80 (4.75)	3.72 (4.65)	3.92 (4.57)	4.72 (4.89)	6.52 (6.18)
Period length, mean (SD)								
Mean period length	5.20 (0.92)	5.28 (0.86)	5.20 (0.94)	5.13 (0.96)	5.08 (0.98)	5.06 (1.01)	5.07 (1.04)	5.13 (1.10)
Standard deviation of period length	0.65 (0.64)	0.64 (0.64)	0.65 (0.63)	0.65 (0.63)	0.66 (0.64)	0.70 (0.67)	0.76 (0.74)	0.87 (0.84)
Irregular cycles, n (row %)								
Irregular	3,438,506 (18%)	1,770,289 (20%)	616,498 (16%)	415,943 (14%)	283,846 (14%)	217,357 (18%)	118,163 (29%)	16,410 (45%)
Regular	15,828,067 (82%)	6,987,056 (80%)	3,205,623 (84%)	2,532,059 (86%)	1,771,949 (86%)	1,017,043 (82%)	294,598 (71%)	19,739 (55%)

### Cycle length

**Figure 1:**
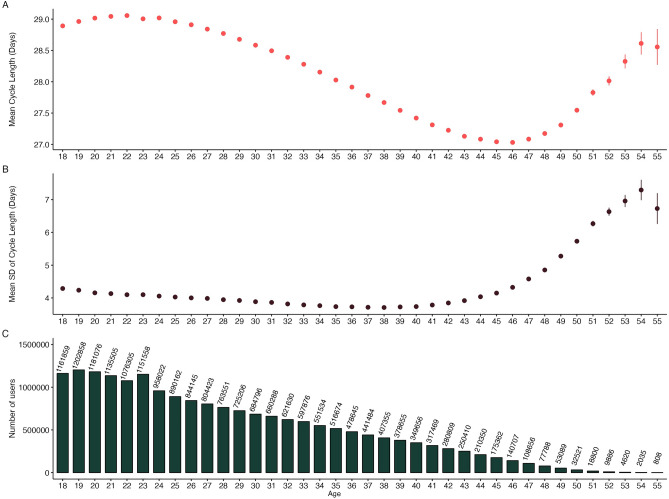
(**A**) Mean cycle length and (**B**) variation in cycle length as measured by standard deviation of cycle length and (**C**) counts of number of users by age.

A one way ANOVA revealed that cycle length differed between age bands (F = 111,288.8, ges = 0.033). Using pairwise tests, we found that mean cycle length shortens from 28.54 days in the 18–25 year old age band to 27.15 days in the 46–50 year old age band. (Mean difference = 1.85 days, t = 363.19, Cohen’s d = 0.59) (Table 1). However, cycle length tends to increase after age 45, with the mean cycle length being longer in the 51–55 age group compared to the 46–50 age group (Mean difference = 0.86 days, t = 48.85, Cohen’s d = 0.26). A similar, non-linear pattern of cycle length differences is observed when plotting mean cycle length by year of age (Fig. 1A). Mean cycle length increases from age 18 to around 21–22, when cycle length is the longest. Between the ages of 22 and 45, cycle length decreases to its shortest, before increasing again post age 45.

Cycle variability, as measured by the standard deviation of cycle length for each user across the 12-month study period, also shows age-associated differences (F = 6830.59, p < 0.001, ges = 0.002) (Table 1). In 18–25 year olds, the mean standard deviation of cycle length is 4.14 days, which is 1.1 times higher than the mean standard deviation of cycle length in the 36–40 year olds (Mean SD = 3.72 days, Mean difference = 0.42 days, t = 114.63, Cohen’s d = 0.09). Compared to the minimum variability in the 36–40 age group, the oldest age group shows higher variability, at 6.52 days (Mean difference = 2.80, t = 111.43, Cohen’s d = 0.51). This pattern of gradually lower cycle variability from 18 to 37 years, before higher variability from the age of 37 onward is also seen when mean cycle variability is plotted by age (Fig. 1B). Figure 1C indicates sample sizes by year of age.

The proportion of users with irregular cycles was highest in the 51–55 age band (44.7%), followed by the 46–50 age band (28.3%). Irregular cycles were least common in the 36–40 age (13.8%) band (Table 1).

### Period length

**Figure 2:**
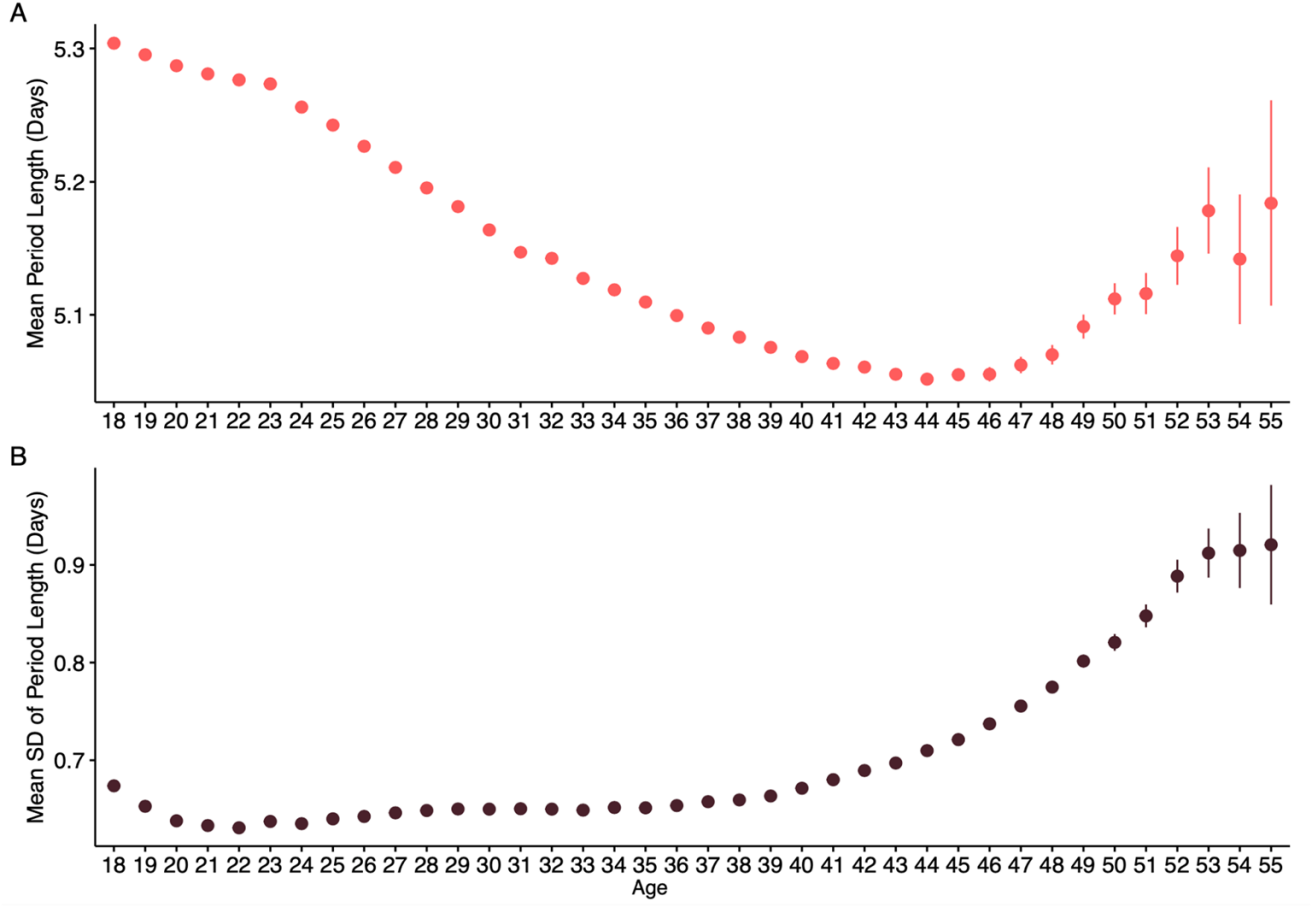
Plots of (**A**) mean period length and (**B**) variability in period length, as measured by standard deviation length, by age.

The mean duration of periods experienced also differed by age (F = 25,167.33, ges = 0.008), with the longest periods in the youngest and oldest age groups. Periods are longest in the 18–25 age group, with a mean duration of 5.20 days. Mean period length then lowers with advancing age to a minimum in the 41–45 age group, at 5.06 days (Mean difference = 0.22, t = 249.17, Cohen’s d = 0.24). However, mean period length becomes longer in the 51–55 year olds, compared to the 41–45 year old participants (Mean difference = 0.08, t = 15.6, Cohen’s d = 0.07). This U-shaped relationship is also revealed when mean period length is plotted by year of age. Figure 2A shows that mean period length is highest in 18 year olds, but gradually lowers until age 44, whereafter period length increases again in users aged 51–55.

Variability in period length, as measured by the standard deviation of period length for each user across the 12-month study period also differed by age group (F = 4161.85, ges = 0.001). The standard deviation of period length was lowest in users aged 26–30, and highest in the 51–55 age group (Fig. 2B). The mean difference between the 18–25 age group and 51–55 age group is 0.23 days (t = 67.81, Cohen’s d = 0.31). This pattern is also seen when period variability is plotted by year of age. Figure 1B shows that variability in period length becomes smaller between 18 and 20 before becoming larger post age 20.

### Most frequent symptom logs by age

A total of 12,392,617 users had logged any of the symptoms of interest in the study time period. Out of the users that logged a symptom 63,482/12,392,617 (0.05%) were removed for an excessive number (more than five times the standard deviation of the mean of the total logs per user) of symptoms. This left a total of 12,329,135 users who were included in the symptom level analysis.

**Figure 3:**
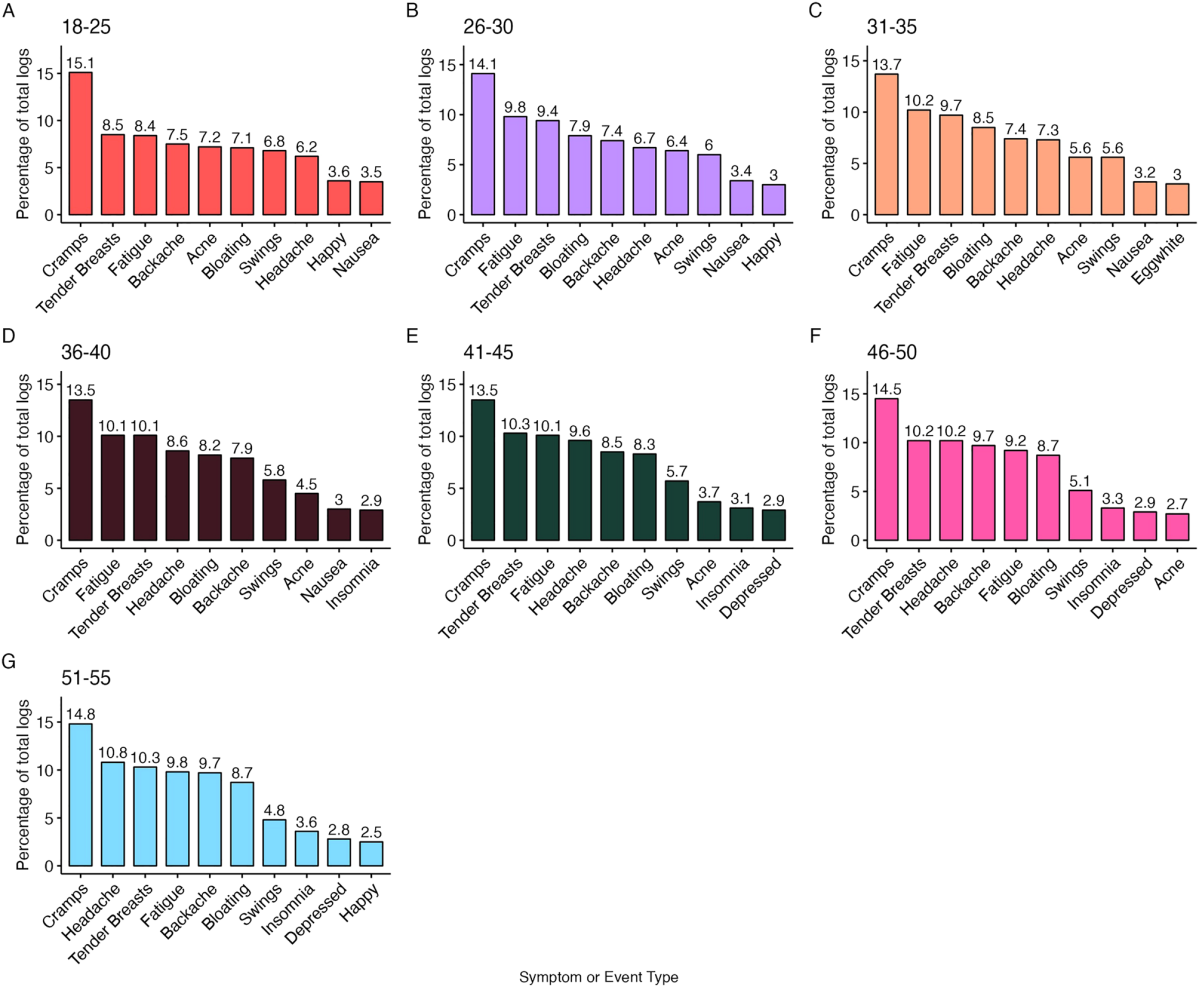
Percentage of total symptom logs by age band.

The frequencies of symptom logs are presented in Supplementary Table 1. The top 10 symptoms logged by each age group are presented in Fig. 3. For the 18–25, 26–30, 31–35, 36–40 and 41–45 age groups, the top three symptoms logged were cramps, breast tenderness and fatigue, though in the 26–30, 31–35 and 36–40 age groups fatigue was slightly more common than breast tenderness. In the 46–50 and 51–55 age groups, cramps, breast tenderness and headache were the top three most common symptoms logged.

### Frequency of symptoms

**Figure 4:**
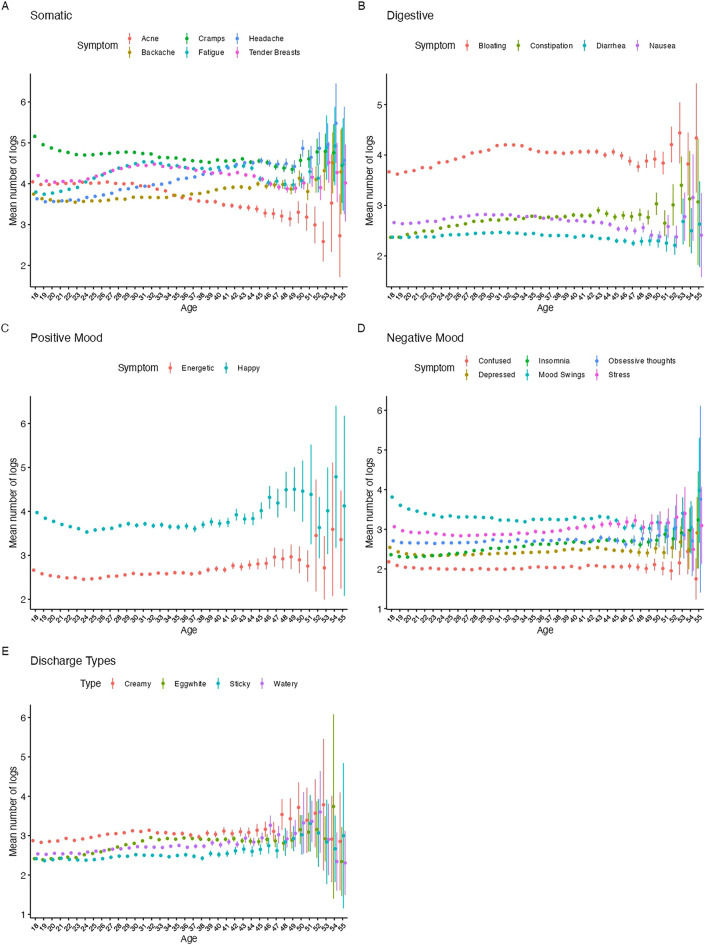
Mean number of symptom logs by age for (**A**) somatic, (**B**) digestive, (**C**) positive mood, (**D**) negative mood, and (**E**) vaginal discharge*.*

We assessed the mean number of logs by age for six somatic symptoms (Cramps, Tender Breasts, Headache, Fatigue, Backache and Acne) (Fig. 4A). While logs of cramps and acne tend to decrease in frequency with advancing age, reports of headache and backache tend to get more frequent in older participants. In contrast, reports of tender breasts and fatigue peak in the late twenties and early thirties.

For digestive symptoms (Fig. 4B), including bloating, constipation and diarrhea, there is little difference in the frequency of logging at different ages, but there is a larger number of logs of constipation at older ages, while overall, logs of bloating are 3.6 times higher than for constipation, 2.6 times higher than Diarrhea and 2.3 times higher than Nausea.

Mood symptoms also tend to remain fairly constant, albeit with a higher number of logs of happy or energetic mood at age 18 than in the 30–35 year olds. Logs of energetic mood are less frequent in older users, after age 49, and logs of happy mood peak around age 50 (Fig. 4C). Overall negative mood logs also show little differences with age, though reports of mood swings tend to be highest in the youngest and oldest users, while stress and insomnia logs are higher in older than younger users (Fig. 4D).

Finally, differences in vaginal fluid logs are seen between different ages. Logs of creamy, watery and sticky fluid remain constant until around age 47–48, where they become more frequent and variable. The frequency of egg white fluid logs show a peak from 32–47, before becoming variable after age 50 (Fig. 4E).

## Discussion

In this paper, we present the largest study to date to investigate temporal characteristics of the menstrual cycle and self-reported symptom frequencies for a sample of over 19 million women aged 18–55. We found patterns of menstrual cycle and period length shortening and increasing variability with advancing age and demonstrated age-dependent patterns in self-reported menstrual cycle-related symptoms.

The menstrual cycle does not remain static over the reproductive lifespan. Existing research that has investigated the cycle in large epidemiological samples, or mHealth samples, has clearly demonstrated that the cycle tends to get shorter, and more variable, with increasing age^37,38^. Broadly, these findings are replicated in this study, but the larger sample size and wider age range used in this study allow for additional insights. Previous studies have demonstrated that cycle length increases between ages 18–23, before beginning a linear decrease in cycle length between the ages of 23 to 45^39^. But, here we can additionally demonstrate that after age 47 cycle length increases sharply until age 55, with a corresponding increase in cycle variability and cycle irregularity. Clinical guidelines are often structured around a median menstrual cycle length of 28 days, with the timing of ovulation being approximately centered at around cycle day 14, and overall, the mean cycle lengths presented here agree with these guidelines. However, the results presented here also show that there is significant age-associated variability in cycle length, particularly around the ages associated with perimenopause.

Similar to cycle length, the duration of bleeding days tends to decrease from a maximum in users aged 18 to a minimum around age 45, after which bleeding days length tends to increase. This pattern is mirrored by a general trend of increasing variability in bleeding days length with age. This increase in cycle and period length variability after age 45 is likely due to the menopausal transition, where cycles tend to become more variable. While the transition to menopause is generally associated with shorter cycles, there is evidence that cycle length actually increases near to menopause^5^, which is supported by our current findings, and is not shown in previous large mHealth studies investigating the menstrual cycle over narrower age bands^38,39^.

Menstrual cycle-related symptoms may differ in intensity or may even appear or disappear at different stages of the reproductive life span. Overall, the pattern of symptoms most frequently logged by users aged 51–55 is quite different from the pattern logged by users in the 18–25 age band, with a greater number of logs for headache, fatigue, and backache for example in older users compared to younger users. These differences in the symptoms most frequently logged are also mirrored by distinct age-related patterns in logging at an individual symptom level. A symptom like acne, which shows a general decreasing trend in logging with older age has a different pattern to headache, which shows a general increase in logging with age, and both display a different pattern compared to symptoms such as happy mood or energetic mood, which are most frequently logged in the youngest and oldest participants. Understanding these patterns is important in ensuring that both individuals understand when to seek treatment and that clinicians understand when treatment is required.

It is notable that around the age of 47, there are marked changes in cycle length and variability, along with changes in the frequency of symptom logs, with many symptoms showing a smaller or greater number of logs. For example, a decrease in bloating, nausea and negative mood logs such as stress, is mirrored by increases in energetic and happy mood logs and increased variability in vaginal discharge logs. While these differences in the frequency of logging of these symptoms around this age are clearly demonstrated and are likely signs of the menopausal transition, they should be viewed in the context of a continuous change in the level of symptoms experienced over the lifespan. Traditionally, menopause has been viewed as a discrete period of change, but there are likely precursor changes that can be observed long before a woman knows they are perimenopausal.

Many menstruators feel that they do not have enough information about the menopause^19^, and a large proportion of individuals experience symptoms related to the menopause that are severe enough to lead them to seek out medical intervention^13–15^. Therefore, the results presented here are important in informing individuals about both the transition to menopause, what to expect, and how the menstrual cycle changes over the lifespan. Understanding these differential experiences, and differences in what is normal at different life stages is important in a wide range of scenarios, from medical care, daily life, and family life. Maintaining health literacy through education, including through applications like Flo is important in ensuring that individuals know when they need medical attention, and for maintaining quality of life and satisfaction^22,40,41^.

Strengths of the current study include being the largest study of its kind, a novel analysis of age-related menstrual cycle symptoms and patterns across the reproductive life span, and most importantly, the inclusion of data from over 400 thousand participants between the ages of 46 and 55, providing important information about the experiences of aging menstruators, who are often excluded from similar research.

However, the study does have limitations. Firstly, when reporting the start and end of a period, a user is initially offered to enter a duration of five days, but can change this duration. This may lead to a bias towards 5 day duration periods in the dataset. Secondly, it is known that ethnicity can affect the menstrual cycle. For example, individuals from Asia have an earlier onset of menopause on average than individuals from Europe^42^. As part of Flo’s commitment to privacy, ethnicity information is not collected or stored by the app, meaning we are unable to investigate how ethnicity might change frequency of symptom logging, or cycle characteristics in this sample. While we excluded users who had set reminders to take contraception, we cannot be sure that all users included in the study are not taking hormonal contraception that could alter the cycle and symptoms. Similarly, we are not able to identify any users who are receiving hormonal therapy due to menopausal symptoms. Also, we were not able to investigate frequency of logging of some symptoms that may be particularly related to perimenopause and menopause, such as hot flashes, night sweats, or brain fog, since these symptoms are not available to log in the app. This, along with less frequent periods may mean that women who are experiencing the menopausal transition may be less inclined to engage with the app. This likely contributes to the age imbalance in our sample, where while we include over 1.5 million users older than 40 in our sample, the majority of participants are aged 18–25. Any further research should endeavor to include classic perimenopausal and menopause symptoms, along with extra information such as ethnicity and, if possible, lab-based measurement of blood markers such as antimüllerian hormone, inhibin-B, and follicle stimulating hormone to obtain objective markers of ovarian reproductive aging. This would allow for a better interpretation of how symptoms relate to ovarian aging, perimenopause, and menopause. Finally, it is also important to note that some of the effects found, such as the effect of age on cycle variability in pre-menopausal individuals and for age on period length during the menopausal transition, have very small effect sizes (< 0.2) and may not represent meaningful differences between groups.

## Conclusion

Here we present the results of the largest study using the broadest age range to investigate self-reported menstrual cycle length, variability and associated symptoms in a sample of menstruating Flo *app* users aged 18–55. We demonstrate clear age-related trends in menstrual cycle and period length, in variability in these cycle indices as well as in symptom spectrum across the entire age range that spans from premenopause into the menopause transition. These patterns and differences in symptoms experienced are important in understanding the menstrual cycle at different life stages, and should be incorporated into educational and medical resources to better inform women about the menstrual cycle.

### Supplementary Information

Supplementary Table 1.

## Data Availability

The data that support the findings of this study are available from Flo Health UK Limited, but restrictions apply to the availability of these data, so they are not publicly available. Data are however available from the authors upon reasonable request and with the permission of Flo Health UK Limited.
